# Evaluating the Efficacy of Cervical Erector Spinae Plane Block Using Ultrasound Versus Fluoroscopic Guidance for Cervical Pain: A Case Series

**DOI:** 10.5812/aapm-160776

**Published:** 2025-05-12

**Authors:** Poupak Rahimzadeh, Sajede Salehi, Sara Saadat, Mahshid Vaziri, Payam Houshyar Azar, Monireh Faghir Ganji

**Affiliations:** 1 Pain Research Center, Department of Anesthesiology and Pain Medicine, Iran University of Medical Sciences, Tehran, Iran; 2 Department of Anesthesia and Pain Medicine, Baqiyatallah University of Medical Sciences, Tehran, Iran; 3 Department of Anesthesia and Pain Medicine, Iran University of Medical Science, Tehran, Iran; 4 Department of Anesthesia, Pain Research Center, Ahvaz Jundishapur University of Medical Sciences, Ahvaz, Iran; 5 Department of Epidemiology, School of Public Health, Iran University of Medical Sciences, Tehran, Iran

**Keywords:** Axial Neck Pain, Case Series, Erector Spinae Plane Block, Fluoroscopic Guidance, Neck Disability Score, Ultrasound Guidance

## Abstract

**Background:**

The erector spinae plane block (ESPB) has traditionally been performed under ultrasound guidance, while fluoroscopic guidance has emerged as an alternative approach.

**Objectives:**

This study aims to compare the efficacy of ESPB using ultrasound and fluoroscopic guidance in patients with cervical pain.

**Methods:**

This case series study includes fourteen patients with axial neck pain scheduled for cervical ESPB. According to the approach of ESPB (ultrasound or fluoroscopic guidance), patients were divided into two groups: Eight underwent ultrasound-guided ESPB, and six received fluoroscopy-guided ESPB. Pain and disability were assessed using the Numerical Rating Scale (NRS) and the Neck Disability Index (NDI) at baseline (pre-procedure), two weeks post-procedure, and three months post-procedure.

**Results:**

Both groups demonstrated significant improvements in NRS and NDI scores over time (P = 0.005). However, no statistically significant differences were observed in pain scores or disability indices at any of the evaluation points.

**Conclusions:**

This study suggests that fluoroscopy-guided ESPB is as effective as ultrasound-guided ESPB for managing cervical radicular pain, providing a viable alternative for clinicians.

## 1. Background

The erector spinae plane block (ESPB) is a novel fascial plane block first described in 2016 and has since been effectively utilized in the management of acute and chronic pain (1). Due to its extensive area of analgesic coverage, ESPB has found applications across various anatomical regions, from the cervical spine to the femur (2-5). In this study, we focused on cervical ESPB to evaluate the efficacy of two different imaging approaches. Although the exact mechanism of ESPB-induced pain relief remains unclear, it is hypothesized that the spread of local anesthetic to the ventral and dorsal rami, paravertebral space, and epidural space contributes to its analgesic effects (6). Traditionally, ESPB has been performed under ultrasound guidance, which allows for real-time visualization of anatomical structures. However, there are certain circumstances in which performing an ultrasound-guided block can be challenging; for example, in patients who are obese or have a short neck, making it difficult for the pain practitioner to obtain a clear view and causing discomfort for the patient. Recently, fluoroscopic guidance has also been demonstrated as an effective alternative for performing ESPB (7, 8). Despite the demonstrated utility of both methods, no studies to date have directly compared the efficacy of ultrasound-guided versus fluoroscopy-guided ESPB. In this case series, we present 14 patients with axial neck pain unresponsive to conservative treatments who underwent cervical ESPB.

## 2. Objectives

The aim of this study is to assess and compare the efficacy of ultrasound-guided and fluoroscopy-guided cervical ESPB in terms of pain relief and functional improvement.

## 3. Methods

Between April 13 and September 16, 2024, patients presenting to our pain clinic with axial cervical pain unresponsive to conservative and medical therapy for at least three months, and classified as American Society of Anesthesiologists (ASA) physical status I or II, were selected for cervical ESPB. Eight patients underwent ultrasound-guided ESPB (U), and six patients received fluoroscopy-guided ESPB (F), and were included in the study. It is noteworthy that patients scheduled for cervical ESPB with a history of cervical spinal surgery (e.g., post-laminectomy syndrome), malignancy, substance addiction, mental disorders, or an ASA physical status classification greater than II were excluded.

### 3.1. Procedure

In the pain operating room, each patient was positioned prone with a pillow placed under the chest to achieve cervical spine flexion. Standard monitoring, including electrocardiography (ECG), noninvasive blood pressure measurement, and pulse oximetry, was applied. The posterior cervical and upper thoracic regions were then aseptically prepared and draped prior to the procedure.

For patients undergoing the ultrasound-guided block, a high-frequency linear ultrasound transducer (5 - 13 MHz, Sonosite S-Nerve, USA) enclosed in a sterile sheath with a thin film of ultrasound gel was positioned sagittally to identify the C7 spinous process. The transducer was then slid laterally to visualize the transverse process of C7. Once the tip of the C7 transverse process was confirmed in the parasagittal plane, a 22-gauge, 90 mm spinal needle (Disposable Spinal Needle, Dr. Japan Co., Ltd., Tokyo, Japan) was introduced using an in-plane approach in a caudal-to-cephalad direction. The needle was advanced toward the tip of the transverse process. After contacting the bone and confirming negative aspiration, hydro-dissection was performed using 2 mL of saline. Subsequently, 15 mL of 0.2% ropivacaine (Ropivacaine Hydrochloride, Bioindustria L.I.M., 5 mg/mL, Italy) combined with 40 mg of triamcinolone (Triamcinolone, CBCORT 40 mg 1 ml, Chandra Bhagat Pharma, India) was injected on each side. The same procedure was implemented for the opposite side.

For patients undergoing the fluoroscopic-guided block, an anteroposterior (AP) view with a slight caudal tilt was obtained to visualize and confirm the C7 vertebral body. The transverse process of C7 was then identified and marked under fluoroscopic guidance. A 22-gauge, 90 mm spinal needle (Disposable Spinal Needle, Dr. Japan Co., Ltd., Tokyo, Japan) was advanced toward the tip of the C7 transverse process. Once the needle contacted the bone and negative aspiration was confirmed, 2 mL of Visipaque contrast (VISIPAQUE 320 mg I/mL, 50 mL vial, GE Healthcare AS, Oslo, Norway) was injected to verify the spread of the contrast within the plane of the erector spinae muscle under coaxial fluoroscopic view. Following this confirmation, 15 mL of 0.2% ropivacaine combined with 40 mg of triamcinolone was injected. The same procedure was conducted for the opposite side. Post-injection, another fluoroscopic image was obtained, demonstrating contrast spread extending to the C3 level.

After the procedure, the needle was removed, and patients were transferred to the recovery room. In recovery, patients were monitored for two hours and subsequently discharged.

After obtaining informed consent from all patients, pain and disability were evaluated using the Numerical Rating Scale (NRS; 0 = No pain, 10 = Worst pain imaginable) and the Neck Disability Index (NDI; 0 - 5 = Mild, 6 - 15 = Moderate, 16 - 25 = Severe, > 26 = Very severe). Assessments were conducted at baseline (NRSB and NDIB), two weeks post-procedure (NRS2 and NDI2), and three months post-procedure (NRS3 and NDI3).

Statistical analyses were conducted using STATA version 17, with a 95% confidence interval applied for all tests. Descriptive statistics are presented as means and standard deviations (SD) for continuous variables, while categorical variables are expressed as frequencies and percentages. The chi-square test was used to analyze categorical data. For continuous parametric data with a normal distribution, independent *t*-tests were employed. NRS and NDI outcomes were assessed using repeated measures analysis of variance (ANOVA). A two-way repeated measures ANOVA was performed to evaluate changes from baseline at each time point, both within and between groups. Statistical significance was set at a P < 0.05.

## 4. Results

In this study, 14 patients undergoing cervical ESPB using two different approaches were evaluated in either the fluoroscopic (F) or ultrasound (U) guidance group. Table 1 compares the demographic parameters of the two study groups. As shown in Table 1, there were no significant differences in baseline demographic data between the two groups (P > 0.05).

**Table 1.** Demographic Data and Descriptive Statistics of the Participants ^a^

Variables	Fluoroscopic Guidance	Ultrasound Guidance	Test	P-Value
**Gender**			1.16 ^b^	0.280
Male	28.6	71.4		
Female	57.1	42.9		
**Quantitative variable ** ^**c**^				
Age	61.50 ± 15.28	58.75 ± 21.99	0.261	0.798
Weight	71.83 ± 9.78	66.25 ± 6.45	1.29	0.221
NRS base	9.50 ± 0.837	9.13 ± 1.808	0.468	0.648
NDI base	29.83 ± 7.4	36.88 ± 6.2	-1.930	0.078
NRS 2nd (wk)	4.50 ± 2.9	5.25 ± 3.8	-0.394	0.70
NDI 2nd (wk)	15.33 ± 5.7	25.50 ± 12.87	-1.793	0.07
NRS 3rd (mo)	6.00 ± 3.40	4.0 ± 3.2	1.044	0.321
NDI 3rd (mo)	17.33 ± 5.8	18.33 ± 11.70	-0.187	0.85

Abbreviations: NRS, Numerical Rating Scale; NDI, Neck Disability Index.

^a^ Values are expressed as (%) or mean ± SD.

^b^ Chi-square.

^c^ Independent *t*-test.

**Figure 1.**
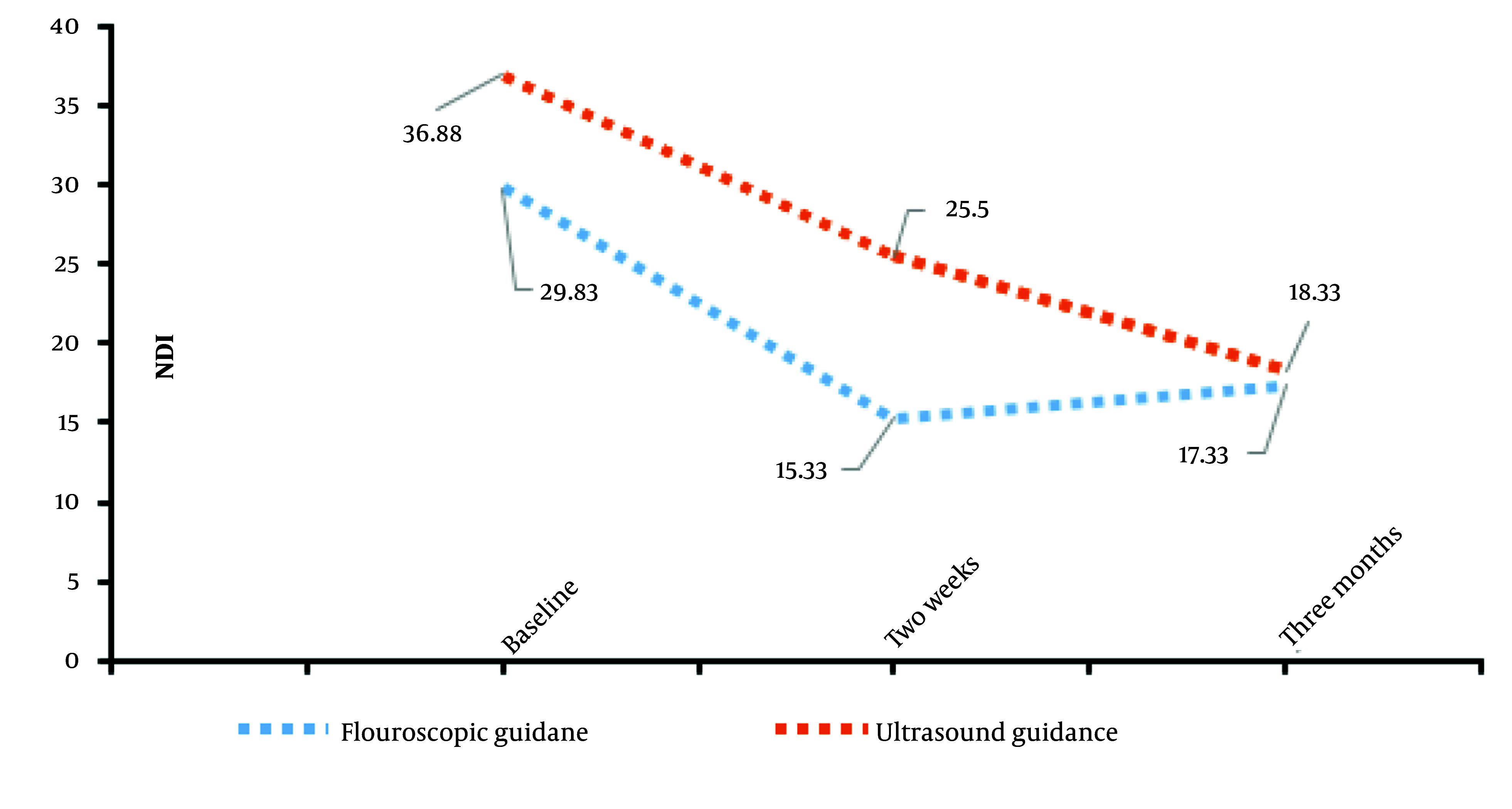
Mean Neck Disability Index (NDI) at three time points: Baseline (0), after two weeks, and after three months, in the two groups are shown.

An ANOVA test was used to analyze differences in NDI scores between the groups at baseline (NDIB), two-week post-procedure (NDI2), and three-month post-procedure (NDI3). The results are reported in Table 2. Referring to Table 2, for NDIB, the F-value was 3.725 with a P-value of 0.078, and for NDI2, the F-value was 3.214 with a P-value of 0.098. While these P-values do not indicate statistical significance, they suggest that with a larger sample size, these differences may become meaningful. For NDI3, the F-value was 0.035 with a P-value of 0.855, indicating no significant difference between the groups even at a threshold of P = 0.1 (Figure 1). 

**Table 2.** Descriptive Statistics and the Results of Analysis of Variance Test for Two Methods (Fluoroscopic Guidance and Ultrasound Guidance) Across Three Variables: NDIB, NDI2, and NDI3 and Three Variables: NRSB, NRS2, and NRS3

Variables	No.	95% Confidence Interval for Mean		F-Value	P-Value
		Lower Bound	Upper Bound		
**NDIB**				3.725	0.078
Fluoroscopic guidance	6	22.05	37.61		
Ultrasound guidance	8	31.66	42.09		
**NDI2**					
Fluoroscopic guidance	6	9.34	21.33	3.214	0.098
Ultrasound guidance	8	14.74	36.26		
**NDI3**				0.035	0.855
Fluoroscopic guidance	6	11.19	23.48		
Ultrasound guidance	6	6.05	30.62		
**NRSB**				0.219	0.648
Fluoroscopic guidance	6	8.62	10.38		
Ultrasound guidance	8	7.61	10.64		
**NRS2**				0.155	0.700
Fluoroscopic guidance	6	1.40	7.60		
Ultrasound guidance	8	2.00	8.50		
**NRS3**				1.091	0.321
Fluoroscopic guidance	6	2.43	9.57		
Ultrasound guidance	6	0.62	7.38		

Abbreviation: NRS, Numerical Rating Scale; NDI, Neck Disability Index.

**Figure 2.**
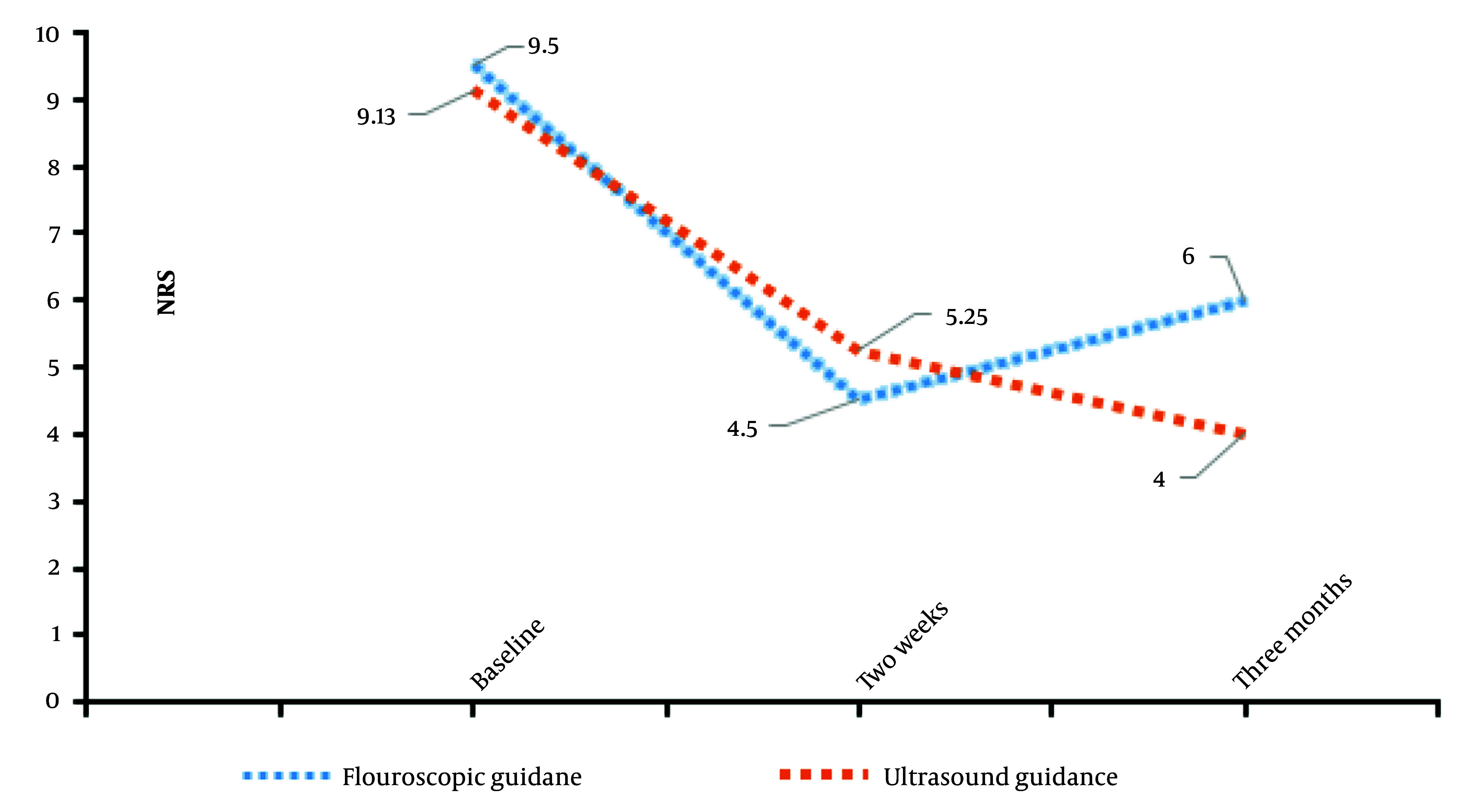
Mean Numerical Rating Scale (NRS) at three time points: Baseline (0), after two weeks, and after three months, in the two groups are shown.

Similarly, ANOVA tests were conducted to evaluate differences in pain scores (NRS) between the groups (Tables 1 and 2). For baseline pain scores (NRSB), the F-value was 0.219 with a P-value of 0.648. For NRS2, the F-value was 0.155 with a P-value of 0.7, and for NRS3, the F-value was 1.091 with a P-value of 0.321. These results demonstrate no statistically significant differences in pain scores at any time point between the two groups (Table 2 and Figure 2). 

The results of repeated measures ANOVA for NRS pain scores within each group revealed significant changes over time. Descriptive statistics indicated that baseline pain scores were significantly higher than those recorded at subsequent time points in both groups. Multivariate analysis further confirmed substantial changes in both NDI and NRS scores across time points, as reflected by significant F-values and P-values. Notable improvements were observed within the first two weeks and were sustained at three months post-procedure. However, the changes between the two-week and three-month assessments were minimal, and drawing conclusions about its long-term or permanent efficacy is not feasible within this timeframe; longer follow-up evaluations are necessary to determine that.

Overall, these findings suggest that both fluoroscopic-guided and ultrasound-guided ESPB approaches are effective in reducing pain and disability over time. However, no significant differences were observed between the two guidance methods in terms of pain or disability outcomes, even with a larger sample size.

## 5. Discussion

The erector spinae muscles are composed of three muscle columns located in the intermediate or deeper layer of the back, running bilaterally alongside the vertebral column. These include the spinalis (the most medial), longissimus (the intermediate column), and iliocostalis (the most lateral). In the cervical region, these muscles are referred to as spinalis cervicis, longissimus cervicis, and iliocostalis cervicis (9). They originate from the spinous and transverse processes and extend laterally, with variable insertion points depending on the cranio-caudal level of the spine (10).

The ESPB is an effective, safe, and straightforward paraspinal fascial plane block that can target the dorsal and ventral rami of spinal nerves at all spinal levels (11). Since its introduction, ESPB has been widely utilized in the management of acute and chronic pain (12). Its mechanism of action is thought to involve the spread of local anesthetic within the fascial plane to block the posterior rami of spinal nerves or anterior spread into the paravertebral space, potentially affecting the sympathetic chain. However, the involvement of the sympathetic chain remains controversial (13-15). Clinical observations suggest that 3 - 5 mL of local anesthetic is sufficient to block a dermatome, and an injection of 30 mL at the T2 level can spread to C3 (16, 17). Based on these findings, we used 15 mL of local anesthetic bilaterally in this study.

Our investigation compared the efficacy of ultrasound-guided and fluoroscopy-guided ESPB in patients with cervical pain. While fluoroscopy-guided procedures are typically performed in the operating room and may incur higher costs compared to ultrasound-guided procedures (which are often office-based), they carry potential risks to the practitioner due to X-ray exposure. However, fluoroscopy can be advantageous in certain patients — such as those who are obese or have a short neck — where performing cervical ESPB under ultrasound guidance is technically challenging. In these cases, obtaining a clear image is difficult, and the required pressure from the probe on the cervical region can be uncomfortable for the patient. Therefore, fluoroscopic guidance may be a preferable option in such patients.

Both groups experienced significant pain relief and functional improvement over three months, with no significant differences between the two guidance techniques. These findings suggest that the choice of imaging guidance may not significantly influence the effectiveness of ESPB for cervical pain.

Previous studies have demonstrated the utility of ESPB in various settings. Prasad et al. successfully performed fluoroscopic-guided ESPB in 31 patients undergoing percutaneous nephrolithotomy for postoperative analgesia at the T8 level (18). Similarly, we demonstrated that fluoroscopic-guided ESPB is effective for managing cervical pain. Patel et al. reported significant pain relief using ultrasound-guided ESPB for postoperative analgesia in 21 patients, aligning with our findings of significant responses to fluoroscopic-guided ESPB (19). Moreover, Robertson et al. showed that ESPB performed under fluoroscopic guidance in 60 patients was effective, consistent with our observation that fluoroscopic-guided ESPB is as effective as ultrasound-guided ESPB (20).

### 5.1. Conclusions

This study highlights the efficacy of the ESPB as a safe and effective technique for managing cervical pain, with significant pain relief and functional improvement observed in both ultrasound-guided and fluoroscopy-guided groups. Our findings suggest that the choice of imaging guidance does not significantly impact clinical outcomes, providing flexibility in selecting guidance techniques based on available resources, practitioner expertise, and especially in selected patients, such as those who are obese or have a short neck with a poor ultrasound view. Further studies with larger sample sizes and extended follow-up periods are needed to validate these findings.

## Data Availability

The dataset presented in the study is available on request from the corresponding author during submission or after publication.
